# The impact of neoadjuvant chemotherapy on the tumor microenvironment in advanced high-grade serous carcinoma

**DOI:** 10.1038/s41389-022-00419-1

**Published:** 2022-07-30

**Authors:** Yuanming Shen, Yan Ren, Kelie Chen, Yixuan Cen, Bo Zhang, Weiguo Lu, Junfen Xu

**Affiliations:** 1 Department of Gynecologic Oncology, Women’s Hospital, Zhejiang University School of Medicine, Hangzhou, Zhejiang China; 2 Women’s Reproductive Health Laboratory of Zhejiang Province, Women’s Hospital, Zhejiang University School of Medicine, Hangzhou, Zhejiang China; 3 Novel Bioinformatics Co., Ltd, Shanghai, China; 4 Zhejiang Provincial Key Laboratory of Precision Diagnosis and Therapy for Major Gynecological Diseases, Women’s Hospital, Zhejiang University School of Medicine, Hangzhou, Zhejiang China

**Keywords:** Ovarian cancer, Cancer microenvironment

## Abstract

High-grade serous ovarian, fallopian tube or peritoneal carcinoma is an aggressive subtype of ovarian cancer that frequently develops resistance to chemotherapy. It remains contested whether the resistance is caused by the acquisition of novel molecular aberrations or alternatively through the selection of rare pre-existing tumor clones. To address this question, we applied single-cell RNA sequencing to depict the tumor landscape of 6 samples from a single case of advanced high-grade serous fallopian tube carcinoma during neoadjuvant chemotherapy (NACT). We analyzed a total of 32,079 single cells, with 17,249 cells derived from the pre-NACT multisite tumor tissue samples and 14,830 cells derived from the post-NACT multisite tumor tissue samples. We identified the diverse properties of the tumor, immune and stromal cell types between the pre-NACT and post-NACT tumors. The malignant epithelial cells displayed a high degree of intratumor heterogeneity in response to NACT. We showed that the primary resistant clone (clone 63) epithelial genotype was already present in the pre-NACT tumors, and was adaptively enriched after NACT. This clone 63 was correlated with a poor clinical prognosis. Furthermore, single-cell analysis of CD4^+^ T cells demonstrated that IL2RAhi-CCL22+-Tregs were selectively enriched in post-NACT tumors. Interestingly, this Treg subtype could recruit and enrich themselves through secreting the CCL22-CCR1 combination in pre-NACT and post-NACT tumors, and further express CD274 to suppress other CD4 and CD8 T cells through a CD274-PDCD1 axis in the post-NACT tumors, and this predicted an immunosuppressive state after NACT. Overall, our results provide important evidence for the adaptive resistance theory of HGSC, and for the potential development of therapeutic strategies to treat HGSC and improve the survival of patients with HGSC.

## Introduction

High-grade serous carcinoma (HGSCs), such as ovarian, fallopian tube, and peritoneal carcinoma are the leading cause of death from gynecologic cancer. As most HGSC cases typically present with multisite peritoneal tumors, it is unclear where the HGSC truly originates. Traditionally, HGSC was hypothesized to arise from the ovarian surface epithelium [1, 2]. Since the late 1990s, accumulating evidence has demonstrated that HGSC is derived from the epithelium of the fallopian tube [3–6]. Multi-region deep sequencing studies and recent single-cell sequencing analyses have shown that these high-grade serous diseases harbor frequent mutations in TP53, high copy-number alterations, and high aneuploidy levels that result in extensive intratumor heterogeneity [7–10].

Recent randomized clinical trials of patients with advanced-stage ovarian, fallopian tube or peritoneal carcinoma have shown that neoadjuvant chemotherapy (NACT) followed by interval debulking surgery (IDS) and primary debulking surgery (PDS) result in similar overall survival (OS) rates in patients [11–13]. Thus, NACT is used increasingly in patients with advanced-stage diseases. While NACT is effective in some patients, it is reported that approximately 70% of patients who undergo NACT, but retain residual viable tumors, have higher rates of recurrence and poor survival prospects [14, 15]. It is hypothesized that residual tumors remaining after NACT contain intrinsically chemo-resistant cell populations [16, 17]. These cell features could differ from the pre-treatment characteristics of the tumor [18–24]. Unfortunately, a large systematic review of 42 studies demonstrated that there are no applicable gene signatures appropriate for clinical use [25]. Furthermore, better treatment responses in advanced stage high-grade serous cancer of the ovarian, fallopian tube or peritoneal carcinoma remains challenging, in part due to the difficulty of obtaining tumors from multiple sites from the same patient, which frequently harbor a distinct tumor microenvironment (TME) [9, 26]. In addition, it remains poorly understood how the complex interaction between tumor cells and the TME affects treatment outcome in these advanced stage high-grade serous diseases. Therefore, there are currently no treatment stratification. The standard of care for these patients who have residual tumors after NACT is the same regimen of carboplatin and paclitaxel as for patients without residual tumors. We hypothesized that comprehensive molecular analyses by comparing pre-treatment tumors and residual tumors after NACT from the same individual, as measured by single-cell sequencing, would assist in identifying innovative therapeutic targets.

In this study, we applied single-cell RNA-sequencing, performing paired analyses of the tumor cells and the TME alterations in pre-NACT and post-NACT samples from the same patient with high-grade serous fallopian tube carcinoma. Importantly, we demonstrated that certain tumor cell clones and CD4^+^ Treg subclusters were adaptively selected to evolve the resistant phenotype in response to NACT. Furthermore, myofibroblastic cancer-associated fibroblasts (myCAFs) and M1/M2 macrophages were enriched in post-NACT tumors, whereas antigen-presenting CAFs (apCAFs), tissue-resident, and glycolysis-related macrophages, and CD8^+^ Naïve T cells were enriched in the pre-NACT tumors. Overall, these findings highlight the characteristics of pre-NACT and post-NACT tumors. This information will offer an opportunity to the development of treatment therapy for patients with advanced high-grade serous diseases.

## Results

### Single-cell transcriptomic analysis in a patient with advanced stage high-grade serous fallopian tube carcinoma treated with neoadjuvant chemotherapy

**Fig. 1:**
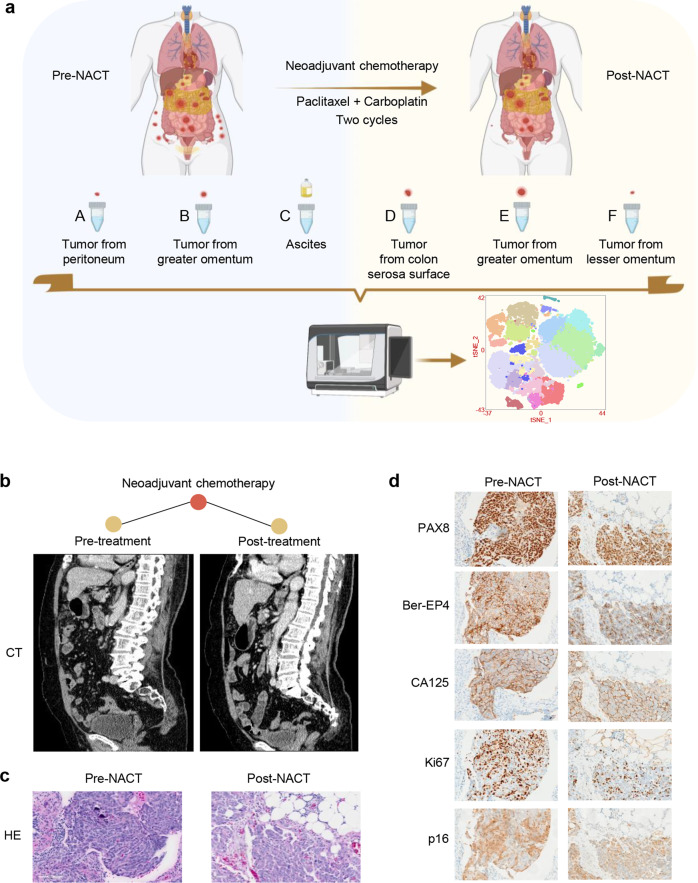
Overview of the treatment schedule and characteristics of an advanced-stage high-grade serous fallopian tube carcinoma. **a** Neoadjuvant chemotherapy treatment and sample acquisition for scRNA-seq. **b** CT imaging showing the clinical responses to NACT of the patient with high-grade serous fallopian tube carcinoma. **c** H&E staining of the pre-NACT and post-NACT samples. **d** IHC staining of PAX8, Ber-EP4, CA125, Ki-67, and p16 protein in pre-NACT and post-NACT tissue samples of the high-grade serous fallopian tube carcinoma.

The analytical scheme following the clinical course of a 71-year-old female patient with stage IIIC high-grade serous fallopian tube carcinoma is summarized in Fig. 1a. The staging and diagnosis of this patient were initially confirmed based on CT imaging plus laparoscopy to evaluate the feasibility of resection and obtain histological biopsy specimens (Fig. 1b, c). We confirmed that this patient was unlikely to be optimally cytoreduced by the assessment laparoscopy. NACT was a better initial treatment option. Then, we collected the pre-NACT tumor samples from the peritoneum and greater omentum, as well as a small volume of ascites (Fig. S1a). After undergoing two cycles of NACT regimens (carboplatin and paclitaxel), the patient received IDS. Dosing per cycle was shown as follows: (1) Cycle 1, carboplatin 535 mg (AUC 5) and paclitaxel 283 mg (175 mg/m^2^); (2) Cycle 2, carboplatin 503 mg (AUC 5) and paclitaxel 284 mg (175 mg/m^2^). We collected the post-NACT tumor samples from the colon serosa surface, greater omentum, and lesser omentum at the time of IDS (Fig. S1a). This patient was characterized as a high-grade serous fallopian tube carcinoma based on the H&E staining, exhibiting strong expression of PAX8, Ber-EP4, CA125, Ki-67, p16, WT1, and mutant p53 protein (Figs. 1c, d and S1b). Genetic risk evaluation showed that this patient did not have a BRCA1/2 mutation in germline or tumor DNA.

**Fig. 2:**
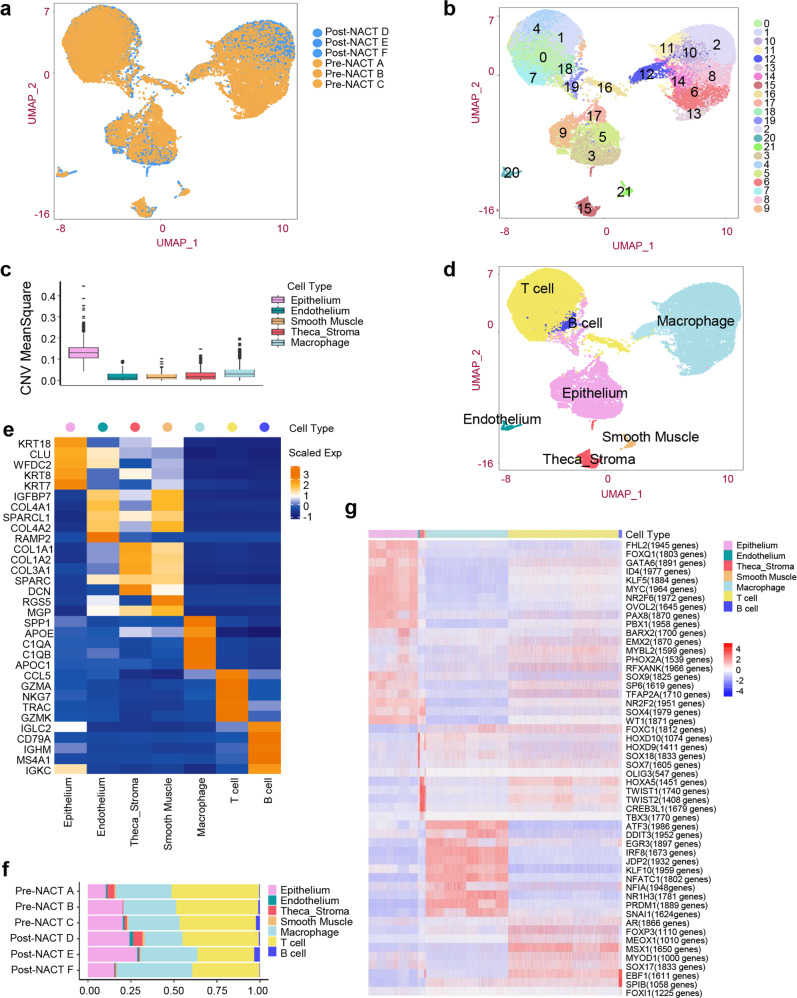
scRNA-seq profiling of the pre-NACT and post-NACT high-grade serous fallopian tube carcinoma samples. **a** UMAP plots showing cell groups by color in the pre-NACT and post-NACT sample groups. **b** UMAP plots showing pre-NACT and post-NACT cells, clustered and color-coded according to the group. **c** Box plots showing the CNV signals for each cell type. **d** UMAP plots showing the cell types by color. **e** Heatmap showing the top marker genes in each cell type. **f** Histogram indicating the proportion of cell types in each analyzed sample. **g** Heatmap showing the expression regulation by transcription factors in each cell type, as estimated using SCENIC. UMAP uniform manifold approximation and projection.

Single cell RNA (scRNA) sequencing using the 10 x Genomics Chromium platform was performed on these matched longitudinal specimens from this patient to assess the cell compositions of the tumor environment in response to NACT. After stringent filtering, a total of 32,079 cells from the six samples, with 17,249 cells derived from the pre-NACT multisite tumor tissue samples and 14,830 cells derived from the post-NACT multisite tumor tissue samples, were retained for further analysis (Fig. 2a). The cell distribution of each sample was shown in Fig. S2. We conducted clustering to define 22 clusters that were visualized using uniform manifold approximation and projection (Fig. 2b and S3a). Copy number variation (CNV) analysis was used to distinguish malignant and non-malignant cells (Fig. 2c). These cells were assigned to seven distinct cell types using known marker genes (Figs. 2d, e and S3b): macrophages (marked with SPP1, APOE, C1QA, C1QB, and APOC1, and MS4A7); T cells (marked with CCL5, GZMA, NKG7, TRAC, GZMK, CD3D, CD3E, CD8A, and CD4); malignant epithelial cells (marked with KRT18, CLU, WFDC2, KRT8, KRT7, and EPCAM); stroma cells (marked with COL1A1, COL1A2, COL3A1, SPARCL1, DCN, and LUM); endothelial cells (marked with RAMP2, VWF, and CLDN5); smooth muscle cells (marked with RGS5 and MGP); and B cells (marked with IGLC2, CD79A, IGHM, MS4A1, and IGKC). Although the proportion of each cell type varied greatly by sample, we found that epithelial cells were enriched in the post-NACT samples, whereas T cells were relatively enriched in the pre-NACT samples (Fig. 2f). Furthermore, we applied single-cell regulatory network inference and clustering (SCENIC) to assess the transcription factors underlying the differences in the expression among different cell types. This identified a set of upregulated transcription factors, such as FHL2, FOXQ1, ID4, KLF5, MYC, NR2F6, OVOL2, PAX8, BARX2, SOX9 in epithelial cells, SOX4 in stromal cells, SOX7 and SOX18 in endothelial cells, ATF3, DDIT3, IRF8, JDP2, KLF10, NFATC1, NR1H3, PRDM1 and SNAI1 in macrophages, FOXP3, MEOX1 and MSX1 in T cells, and SPIB in B cells (Fig. 2g).

### Distinct features and adaptive clonal evolution of malignant cells in response to NACT

**Fig. 3:**
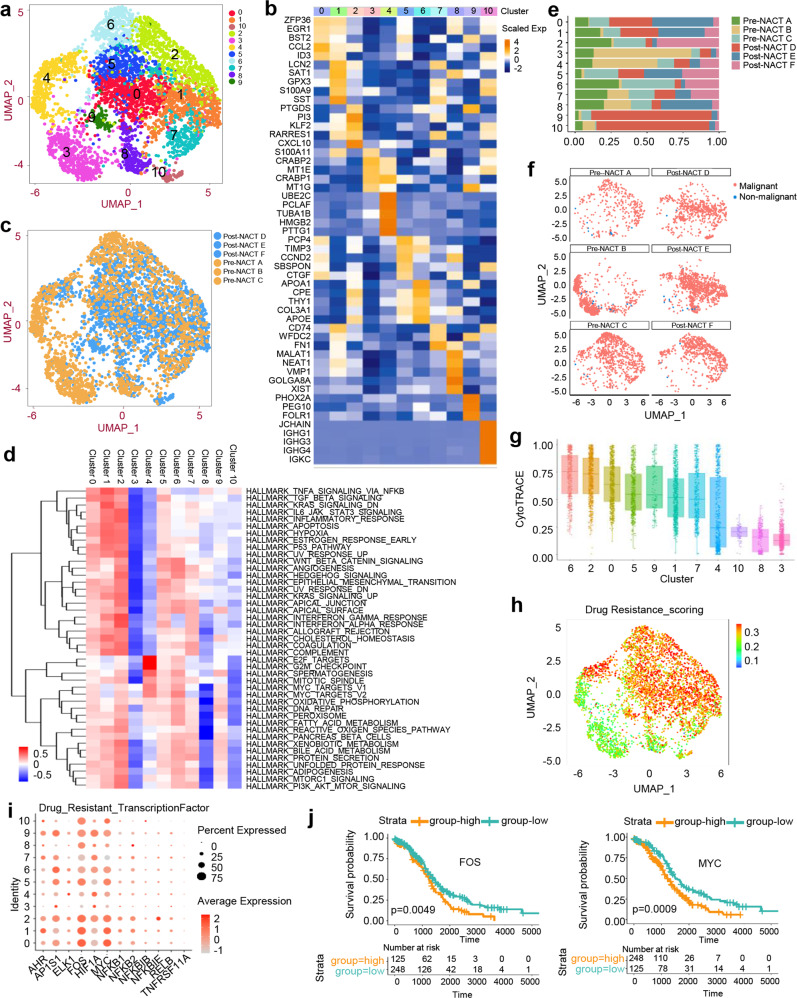
The subtypes of epithelial cells in pre-NACT and post-NACT tumors. **a** UMAP projections of subclustered epithelial cells, labeled in different colors. **b** Heatmap showing the expression of marker genes in each indicated cell subcluster. **c** UMAP projection showing pre-NACT and post-NACT cells, clustered and color-coded according to the group. **d** Heatmap indicating the primary hallmark pathways in each subcluster. **e** Histogram indicating the proportion of subcluster cells in each analyzed sample. **f** UMAP plots showing the malignant and non-malignant epithelial cells colored by red or blue in each tumor sample. **g** CytoTRACE analysis of epithelial cells in each subcluster. **h** UMAP plots showing distinct drug resistance scores in each subcluster. **i** Gene bubble plots showing different expression levels of drug resistant-related transcription factors in each subcluster. **j** Kaplan–Meier survival curves showing the association of FOS and MYC expression with overall survival in patient with HGSCs (from TCGA). Log-rank *p* values are shown.

To define the major subpopulation structure of the malignant epithelial cells, we performed MNN clustering on our scRNA-seq data, identifying 11 main subclusters with a panel of specific marker genes (Fig. 3a, b). We compared each subcluster before and after NACT (Fig. 3c). However, we did not find a new malignant subcluster that conferred a chemoresistant phenotype induced by NACT. All the subclusters were already present in the pre-NACT tumors. Hallmark analysis revealed that subclusters 0, 1, 2, and 5 were enriched in TGF-β signaling, WNT/β-catenin signaling, angiogenesis, and epithelial-mesenchymal transition (EMT) (Fig. 3d). Strikingly, we noticed that the vast majority of four subclusters were derived from post-NACT D, E and F tumor cells (Fig. 3e), suggesting that they might share a resistant genotype. We further observed that these cells from the pre-NACT tumors were primarily detected in ascites (Fig. 3e). The ascites also possessed more malignant epithelial cells compared with those in other tumor samples in the pre-NACT group (Fig. 3f). Moreover, enrichment of stem cells in subcluster 0, 1, 2 and 5 was also identified based on the cytoTRACE analysis (Fig. 3g). We showed that the drug resistance scoring was significantly increased in these subclusters (Fig. 3h). FOS and MYC were determined to be the underlying transcription factors contributing to drug resistance (Fig. 3i). Kaplan-Meier survival analysis showed that higher expression of FOS and MYC in HGSC data obtained from TCGA was associated with shorter OS times (Fig. 3j). Collectively, our findings suggest that chemoresistance may arise due to the selection and expansion of pre-existing subcluster malignant cells.

**Fig. 4:**
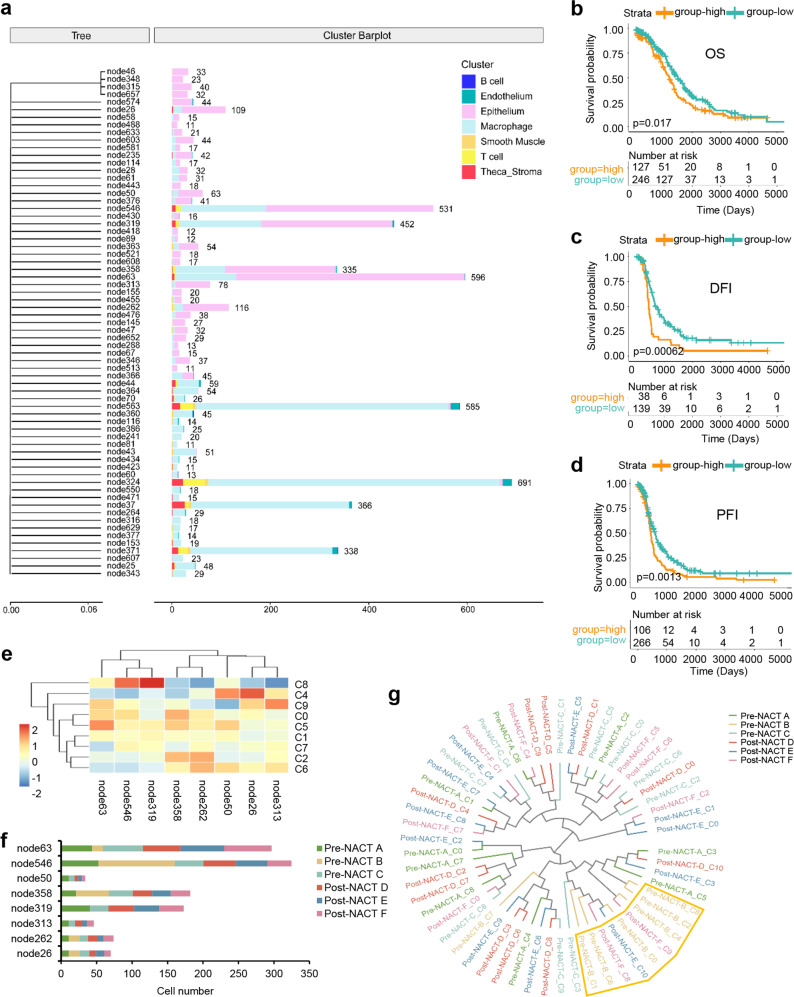
Analysis of clonal evolution in high-grade serous fallopian tube carcinoma tissues following NACT treatment. **a** Phylogenetic trees showing the analyses of the clonal dynamics calculated from each cell type, and the subpopulation-specific differences in each clone are indicated with color-coded bars. Kaplan–Meier survival curves for OS (**b**), DFI (**c**) and PFI (**d**) from TCGA HGSC data showing significant prognostic separation according to the clone 63 marker gene signatures from our scRNA-seq data. Log-rank *p* values are shown. **e** Clonal frequencies of the eight main clone cells are annotated in each epithelial subcluster. **f** The cell number distribution of the eight main clones in each tumor sample. **g** Evolutionary tree analysis showing the distribution of epithelial subclusters and corresponding tissue samples. DFI disease-free interval; PFI progression-free interval.

To confirm the adaptive resistance hypothesis, we further conducted lineage tracing analysis using VarTrix (Fig. 4a). The phylogenetic trees showed four major subclones in malignant cells, including subclone 546, 319, 358, and 63 (Fig. 4a). Among these, analyses of HGSC cohorts from TCGA supported the enrichment of the top 56 marker genes associated with clone 63 cells as a significant indicator of adverse clinical outcomes in HGSC patients (Fig. 4b–d and Table S1). The expression of the gene signature was correlated with a significantly poorer clinical prognosis, including a shorter disease-free interval, progression-free interval, and OS time. Furthermore, the cell subcluster and tissue location distribution of all the clones was analyzed (Fig. 4e, f). The clusters 3 and 10, containing less than 50 epithelial clone cells, were excluded from the analysis. We found that the main clones 63, 546, 319, and 358 were persistent in both pre-NACT and post-NACT samples, and clone 63 was further enriched in the post-NACT samples (Fig. 4f), consistent with adaptive resistance. Of note, we observed that the primary pre-NACT tumor cells in the greater omentum were mostly eliminated in this clone. The tumor cells in the post-NACT greater omentum sample were primarily derived from pre-NACT ascites and the peritoneum tumor cells. In addition, we conducted evolutionary tree analysis based on the information of epithelial subclusters and tissue samples (Fig. 4g). We found that no matter which sample B cells (from pre-NACT greater omentum tissues) were evolved from in clones, they were convergent, whereas other samples had obviously mixed with each other, and there was no sample convergence. From the perspective of the composition of the clones, our present study supports the notion that the cells of post-NACT omentum tumors were not the same as pre-NACT omentum tumors, but more likely originated from the other pre-NACT samples. This may suggest a novel adaptive resistance theory for HGSC.

### Fibroblasts from post-NACT tumors show functional alterations compared with fibroblasts from pre-NACT tumors

Further clustering in the fibroblast compartment gave rise to 3 cell subpopulations (Fig. S4a and b), of which one fibroblast cluster expressed high levels of ACTA2, POSTN, and HOPX, confirming their identity as myCAFs; another cluster expressed major MHC-II genes such as HLA-DRA, CD74, and HLA-DRB1, and was, therefore, termed them apCAFs; and a cluster expressed high levels of CFD, DPT, and CXCL12, and was considered to be inflammatory CAFs (iCAFs) (Fig. S4b). Interestingly, myCAFs and iCAFs were mainly enriched in post-NACT tissues, whereas apCAFs were mainly present in pre-NACT tissues (Fig. S4a). Moreover, apCAFs were related to response to IFN (Fig. S4c and S4d), while myCAFs indicated significant enrichment for WNT/β-catenin signaling (Fig. S4e).

### Macrophage subclusters between pre-NACT and post-NACT tumors

Specific tumor-associated macrophage (TAM) subtypes have important impacts on ovarian cancer progression and therapy [27–29]. For instance, M2-like TAMs limit the effector function of CD8+T cells in metastatic HGSCs and associated with poor overall survival [27]. Thus, we studied the macrophage subclusters in our samples. According to the top differentially expressed genes and known marker genes, macrophage subclusters were designated as tissue-resident-, glycolysis related-, M1-, M2-, and cycling macrophages (Fig. S5a–c). Of note, M1 and M2 macrophages were mainly enriched in post-NACT tissues, whereas tissue-resident and glycolysis-related macrophages were mainly present in pre-NACT tissues (Fig. S5d).

### Features of CD4^+^ Treg subtypes in response to NACT

**Fig. 5:**
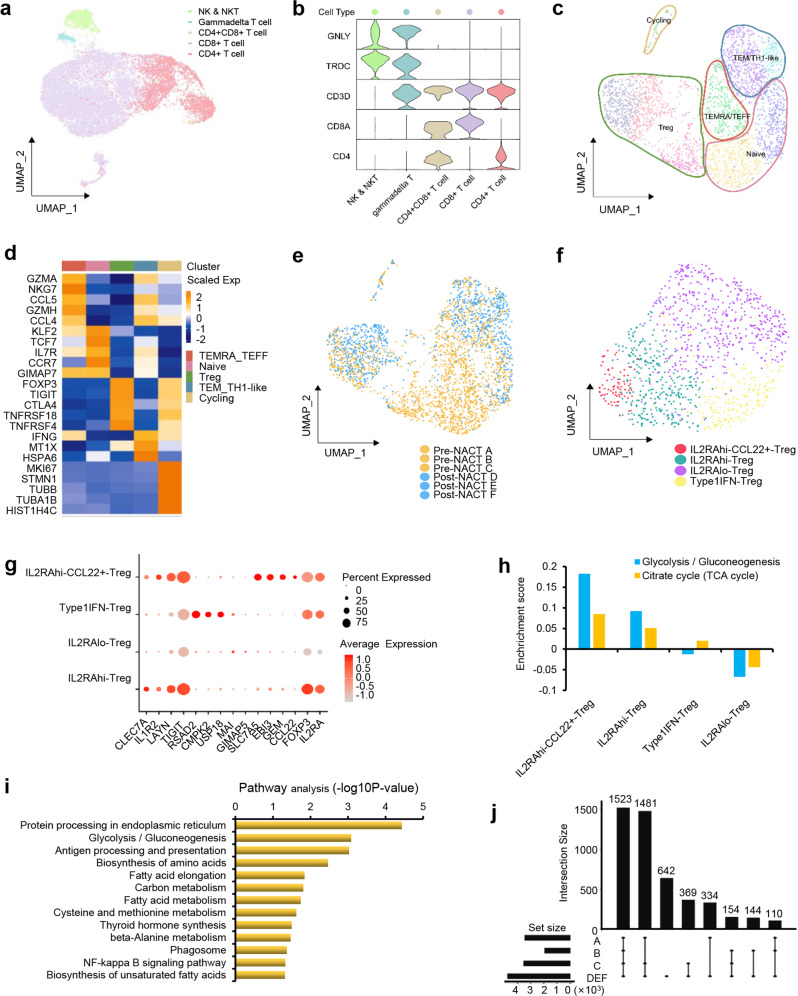
Distinct Treg subpopulations detected in pre-NACT and post-NACT tumors. **a** UMAP plots showing the subtypes of T/NK cells, labeled in different colors. Subtype annotations are indicated in the Figure. **b** Violin plots showing selected marker genes in distinct T/NK cell subtypes. **c** UMAP plots showing the subtypes of CD4^+^ T cells from pre-NACT and post-NACT tumors. Each subcluster is color-coded. Subtype annotations are indicated in the Figure. **d** Heatmap depicting marker gene enrichment for each cell subtype of CD4^+^ T cells. **e** UMAP plots showing the color-coded cell groups of CD4^+^ T cells in response to NACT. **f** UMAP plots showing four main cell subtypes of Tregs, labeled in different colors. **g** Dot plots showing the expression levels of specific genes in each Treg subtype. **h** Metabolic pathway analysis showing the enrichment of Glycolysis/Gluconeogenesis and the citrate cycle in each Treg subtype. **i** KEGG pathway analysis showing the primary enriched pathways of IL2RAhi-CCL22+-Treg cells. **j** Cell communication analysis showing the overlapping relationship between specific pre-NACT and post-NACT tumor samples.

The clustering of T/NK cells of the tumor environment revealed 5 main populations, including NK/NKT subtype (GNLY and TRDC), γδ T cells (GNLY, TRDC, and CD3D), CD4^+^CD8^+^ T cell subtype (CD3D, CD8A, and CD4), CD8^+^ T cells (CD3D and CD8A), and CD4^+^ T cells (CD3D and CD4) (Fig. 5a, b). The CD4^+^ and CD8^+^ T cell infiltration into the stroma and tumor epithelium was further determined by CD4 and CD8 IHC staining (Fig. S6).CD8^+^ T cells were further designated as CD8 TEM, CD8 TEMRA/TEFF, CD8 TRM, CD8 Naïve, CD8 IFN response, and CD8 Cycling cell subtypes, according to their marker genes (Fig. S7a, S7b). All these subtypes were shared across tumors and between pre-NACT and post-NACT samples (Fig. S7c). Of note, CD8 Naïve and CD8 IFN response cells were mainly enriched in pre-NACT samples (Fig. S7c).

We then investigated the CD4^+^ T cell heterogeneity to determine their contribution to NACT responses, and identified 5 main subtypes of CD4^+^ T cells, including CD4 TEM/Th1-like, CD4 TEMRA/TEFF, CD4 Naïve, CD4 Treg, and CD4 Cycling cells (Fig. 5c, d). A high diversity of CD4^+^ T cells was observed between pre-NACT and post-NACT tumors (Fig. 5e). We found that the subclusters of Tregs displayed distinct enrichment in response to NACT (Figs. 5c, e). Thus, we re-clustered CD4 Tregs and further identified four states of Tregs, including IL2RA-high and CCL22 positive subtype (IL2RAhi-CCL22+-Treg), IL2RA-high subtype (IL2RAhi-Treg), Type 1 IFN positive subtype (Type1IFN-Treg), and IL2RA-Low subtype (IL2RAlo-Treg) (Fig. 5f), together consisting 34.3% of the tumor-infiltrating CD4^+^ T cells (Fig. 5c). These four Treg states were mainly distinguished by higher expression of known immune checkpoints IL2RA, TNFRSF4/9/18, and CD27 in IL2RAhi-Treg cells, and plus CCL22 in IL2RAhi- CCL22+-Treg cells (Figs. S8a and 5g). The gene features of each state are shown in Fig. 5g.

Cell metabolism is appreciated as a key regulator of T cell function and fate [30–32]. We noticed an extremely high expression of the glycolytic enzyme lactate dehydrogenase A (LDHA) in IL2RAhi-CCL22+-Tregs (Fig. S8b), which indicates LDHA might affect the function of Tregs. Consistently, previous studies reported that LDHA plays a key role in the altered glycolytic metabolism [33]. Therefore, we hypothesized that the glycolytic metabolism be important for those immune effector cells in the tumor. The metabolic features were further analyzed for those subclusters. As shown in Fig. 5h, when compared with the other three subtypes, Glycolysis/Gluconeogenesis was significantly enriched in IL2RAhi-CCL22+-Treg cells, while Citrate cycle was not notably increased, indicating an hypoxic environment with the high expression of LDHA. In addition, pathway analysis of the IL2RAhi-CCL22+-Treg cells also demonstrated that Glycolysis/Gluconeogenesis was the primarily enriched pathway (Fig. 5i). Interestingly, cell communication analysis indicated that the features of the post-NACT samples D, E, and F were more similar with pre-NACT sample A and C than with sample B (Fig. 5j), indicating that these Tregs were present in the pre-NACT tumors and were primarily derived from ascites and the peritoneum, and persisted in all residual tumor samples after NACT therapy.

### Cell cross-talk between immune cells and tumor microenvironment

**Fig. 6:**
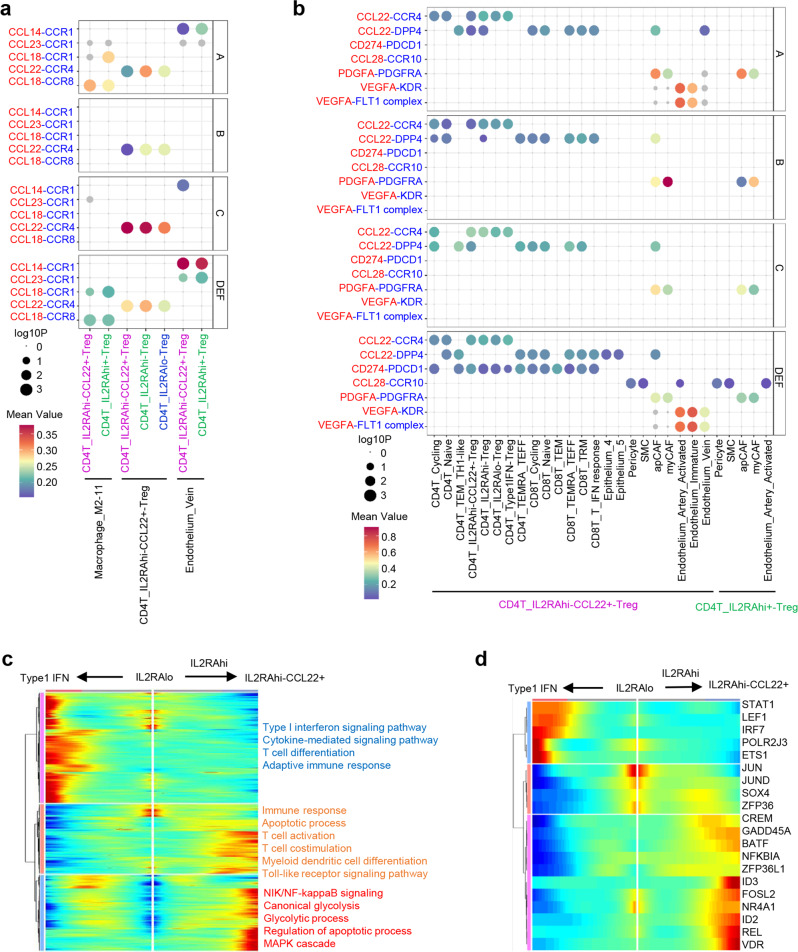
Analysis of cell communication and CD4^+^ Treg cell transition states in pre-NACT and post-NACT tumors. **a** Dot plots showing chemokine-receptor communication between distinct cell types and Treg subtypes in the pre-NACT and post-NACT tumor microenvironment. **b** Dot plots showing ligand-receptor pair analysis of the interactions between IL2RAhi-CCL22+-Treg or IL2RAhi- Tregs and distinct cell types in the pre-NACT and post-NACT tumor microenvironment. **c** Heatmap showing the dynamic changes in gene expression along the pseudotime. The enriched pathways are labeled by colors. **d** Heatmap showing the dynamic changes in transcription factor expression along the pseudotime.

Next, we investigated the correlation between specific Treg subtypes and other cell types to explore the potential functional roles of Tregs. We first conducted cell communication analysis to determine which cell types could affect the enrichment of IL2RAhi-Tregs and IL2RAhi-CCL22+-Tregs (Fig. 6a). Interestingly, we observed that the IL2RAhi-CCL22+-Treg cells could recruit and enrich themselves through secreting the CCL22-CCR1 combination in all tumor samples. We next determined whether the two Treg subtypes could also affect other cell types in HGSC (Fig. 6b). Notably, we observed that these IL2RAhi-CCL22+-Treg cells from the post-NACT D, E, and F samples could express CD274 to suppress other CD4 and CD8 T cells through CD274-PDCD1 axis. Moreover, we found that the IL2RAhi-CCL22+-Tregs from pre-NACT sample A and post-NACT samples D, E, and F could secrete VEGFA to promote angiogenesis of endothelial cells via VEGFA-KDR and VEGFA-FLT1 signaling. In addition, both IL2RAhi-Treg and IL2RAhi-CCL22+-Treg cells could express PDGFA to promote the growth of CAFs through PDGFA-PDGFRA interaction.

### Pseudotime trajectory shows the characterization of distinct CD4+ Treg subtypes

We further explored the dynamic cell transitions by inferring the state trajectories using Monocle (Fig. 6c). This pseudotime analysis revealed that the IL2RAlo-Treg cells were present at the beginning of the trajectory path, whereas the IL2RAhi- CCL22+-Treg cells and Type1IFN-Treg cells were present at the two different terminal stages. One transition was determined to initiate with IL2RAlo-Tregs, through an intermediate IL2RAhi-Treg state characterized by enrichment of immune response, apoptosis, T cell activation, T cell co-stimulation, myeloid dendritic cell differentiation, and toll-like receptor signaling pathway, and finally reach the IL2RAhi- CCL22+-Treg stage, characterized by enrichment of NIK/NF-κB signaling, canonical glycolysis, glycolytic process, regulation of apoptosis, and the MAPK cascade (Fig. 6c). The main branch expression analysis modeling (BEAM) genes for IL2RAhi-CCL22+-Tregs were HAVCR2, CTLA4, TIGIT, TNFRSF4, TNFRSF9, and LAG3 (Fig. S9). The transcription factors enriched in each cell state were further analyzed. As shown in Fig. 6d, we revealed that STAT1, LEF1, IRF7, POLR2J3, and ETS1 contributed to the transition from IL2RAlo-Tregs to Type1IFN-Tregs, while ID3, FOSL2, NR4A1, ID2, REL, and VDR contributed to the transition from IL2RAlo-Tregs to IL2RAhi-CCL22+-Tregs. Collectively, our results suggest that manipulation of Tregs with high immune checkpoint characteristics might present a novel therapeutic strategy for both primary and chemo-resistant HGSCs.

## Discussion

Despite the progress made in treatment strategies, the clinical outcomes of advanced HGSCs remain dismal. The high incidence of early recurrence, resistance to therapeutics, and the lack of efficient therapeutic targets represent the major obstacles in improving OS rates. Recurrent tumors are often treated based on the pathological characteristics of the primary tumor. However, whether the recurrent and primary tumor share a similar microenvironment has not been evaluated. Here, we provide a single-cell transcriptomic atlas to characterize the tumor’s features in pre-NACT and post-NACT samples from the same patient with high-grade serous fallopian tube carcinoma.

A key finding of this study was the identification of chemo-resistant clones in post-NACT tumors. We revealed that the chemo-resistant clones were already present in the pre-NACT tumor cells, and were adaptively selected in response to NACT, consistent with the theory of adaptive resistance. Previous studies have reported that platinum-based chemotherapy-induced new somatic mutations in high-grade serous ovarian cancer, supporting the acquired model of therapy resistance [34]. Our data suggest another model of chemoresistance (adaptive) in the establishment of a resistant tumor phenotype of HGSCs. It may raise the possibility of diagnostic opportunities for examining the chemo-resistant clones in HGSC patients prior to the administration of NACT to predict which patients may benefit from NACT. Furthermore, there may be novel therapeutic strategies to overcome chemo-resistance by targeting the chemo-resistant clones.

Importantly, we have identified distinct states of CD4 Tregs that differed based on the expression levels of IL2RA and immune checkpoint genes, such as TNFRSF4/9/18, CD27, and CCL22. It is important to note that the CD4-IL2RAhi- and CD4-IL2RAhi-CCL22+-Treg subtypes were already present in the pre-NACT tumors and persisted in all residual tumor samples after NACT therapy. Of note, the IL2RAhi-CCL22+-Tregs could recruit and enrich themselves through secreting CCL22-CCR1 pair. These Tregs could also express CD274 to suppress other CD4 and CD8 T cells through a CD274-PDCD1 axis. It is possible that the IL2RAhi-CCL22+-Tregs are responsible for a more potent immunosuppressive state of post-NACT tumors. Our data may assist in identifying more effective therapeutic targets for immunotherapies in chemo-resistant HGSCs.

Furthermore, we demonstrated the presence of 3 CAF subtypes, including apCAFs, iCAFs and myCAFs, of which apCAFs were mainly found in pre-NACT tissues, and both iCAFs and myCAFs were primarily found in post-NACT tissues, revealing that the desmoplastic microenvironment of the HGSCs was highly heterogeneous during NACT treatment. It was reported that apCAFs had the ability to present antigens to T cells and potentially modulate the immune response [35]. In this study, myCAFs were the most prevalent fibroblast subpopulation after NACT treatment. It was reported that certain myCAF subtypes could upregulate CTLA4 and PD1 expression in Tregs and were associated with resistance to immunotherapy [36]. Consistent with this, we also found that the IL2RAhi-CCL22+-Treg subtype was enriched after NACT treatment. Moreover, IL2RAhi-Tregs and IL2RAhi-CCL22+-Tregs expressed PDGFA to promote the growth of myCAFs through PDGFA-PDGFRA interaction. Our data uncovered a possible interaction between specific CAFs and IL2RAhi-CCL22+-Tregs, and their roles in immunotherapy resistance.

In the presence of NACT, we found an enrichment of M1 and M2 polarization, while the proportion of the tissue-resident and glycolysis-related macrophages were significantly decreased. In addition, the main feature of CD8^+^ Naïve T cells was to differentiate into effective cytotoxic T cells after antigen stimulation [37]. These cells are known to be associated with protective immunity and better clinical outcomes [38]. Our data showed that the proportion of the CD8^+^ Naïve T cell subpopulation significantly decreased after NACT treatment. These results support the presence of a chemo-resistant microenvironment in the residual tumors after NACT treatment.

The main limitation of our study is that we analyzed the pre-NACT and post-NACT tumor samples at a single-cell resolution from only one case of patient with high-grade serous fallopian tube carcinoma. Future work will need to be performed in a larger cohort of HGSC patients to validate and explore the mechanistic roles of the chemo-resistant signatures.

Collectively, our single-cell sequencing results provide novel insights into the development of chemo-resistance in HGSCs, and may have potential therapeutic values for HGSCs.

## Methods

### Human tissue specimens

The pre-NACT and post-NACT high-grade serous fallopian tube carcinoma tissue samples were obtained from the Women’s Hospital of Zhejiang University with Institutional Review Board (IRB) approval from the Women’s Hospital of Zhejiang University (approval no. IRB-20210313-R) and conducted in accordance with Declaration of Helsinki. Patients were enrolled after providing written informed consent.

### Isolation of single cells

After surgical resection, fresh tissues were obtained and immediately dissected into fractions for enzymatic digestion into single cells. After digestion, the cells were filtered through a 35 µm cell strainer, followed by enrichment of live single cells, and finally loaded for single-cell sequencing.

### scRNA sequencing and statistical analysis

scRNA sequencing and data analysis were performed by NovelBio Bio-Pharm Technology Co., Ltd. using the NovelBrain Cloud Analysis Platform. We used fastp with the default parameters to filter the adaptor sequence and remove low-quality reads to obtain the clean data [39]. We obtained the feature-barcode matrices by mapping reads to the human genome (GRCh38 Ensemble: version 91) using CellRanger v3.1.0. The down sample analysis was applied according to the mapped barcoded reads per cell of each sample and the aggregated matrix was then obtained. Cells containing over 200 expressed genes and a mitochondria UMI rate below 20% passed the cell quality filtering, and finally mitochondrial genes were removed from the expression table.

We applied the Seurat package (version: 3.1.4) for cell normalization and regression based on the expression table. In order to remove the batch effect, we used the fastMNN function (*k* = 5, *d* = 50, approximate = TRUE, auto.order = TRUE) in the R package scran (v1.10.2) and applied the mutual nearest neighbor method based on the scale data of the top 2000 highly variable genes and sample batch info [40]. Utilizing the graph-based cluster method (resolution was optimized in different sub-clustering results of different cell types), we obtained the unsupervised cell cluster results based on the MNN top 10 principal. The significant marker genes were calculated by the FindAllMarkers function using the Wilcoxon rank sum test algorithm. The cell identification criteria were logFC >0.25, *p* < 0.05, min.PCT > 0.1, and *p*.adj <0.05. To identify the sub-cell type detailed, the clusters of the same cell types were selected for sub-clustering analysis, graph-based clustering, and marker analysis.

### Pseudo-time analysis

The single-cell trajectories analysis was performed using Monocle2 (http://cole-trapnell-lab.github.io/monocle-release) using DDR-Tree with the default parameters. Prior to Monocle analysis, we selected marker genes from the Seurat clustering results and raw expression counts of the cells that had passed filtering. Based on the pseudo-time analysis, BEAM analysis was used for branch fate determined gene analysis, and only significant genes (*q* value <0.01) were selected for visualization, and the top 2000 BEAM genes were selected for functionalized annotation using Gene Ontology (http://geneontology.org/) and KEGG pathway annotation (https://www.kegg.jp/).

### Cell communication analysis

Cell communication analysis was applied to study the cell-cell communication molecule features by utilizing CellPhoneDB, a public repository of ligands, receptors and their interactions [41]. Based on the interaction and the normalized cell matrix achieved by Seurat normalization, the significant mean and cell communication significance was determined (*p* < 0.05).

### SCENIC analysis

SCENIC analysis (pySCENIC, v0.9.5) [42] was applied to assess transcription factor regulation strength based on the 20-thousand motifs database for RcisTarget and GRNboost.

### QuSAGE analysis

We performed QuSAGE (2.16.1) analysis [43] to characterize the relative pathway activation of a given gene set based on the geneset collected from KEGG pathway database, Molecular signatures database (http://www.gsea-msigdb.org/gsea/index.jsp), and immune response gene sets from a referenced article [44].

### Differential Gene Expression Analysis

The function FindMarkers with the Wilcoxon rank sum test algorithm was applied to identify differentially expressed genes. The following criteria were used: logFC > 0.25, *p* < 0.05, and min.PCT > 0.1.

### CNV estimation

Cells defined as theca_stroma, endothelium, SMC, and monocytes were used as references to identify somatic copy number variations using the R package infercnv (v0.8.2). For each cell, the extent of the CNV signal was scored and the mean of squares of CNV values across the genome was then defined. Putative malignant cells were identified as those with CNV signals >0.05 and CNV correlations >0.5.

### Cytotrace

To predict the relative differentiation state of cells, we performed Cytotrace (v0.1.0) analysis based on the expression data in epithelia sub-clustering results [45].

### Drug resistance score and transcription factors analyses

The drug resistance-related genes and the related transcription factors were collected from the gene lists of Human Cancer Drug Resistance & Metabolism PCR Array (Qiagen). Drug-resistant score was further calculated using the Z-score mean value of the expressed genes.

### Lineage tracing based on mitochondrial mutation

We used VarTrix (https://github.com/10XGenomics/vartrix) to calculate the alternative allele frequency and the coverage of each position in the mitochondrial chromosome. Then, we built phylogenetic trees based on the mitochondrial mutations clone info following Zhang et al. [46].

### Immunohistochemistry (IHC)

IHC was performed on a 4-μm thick paraffin-embedded human high-grade serous fallopian tube carcinoma tissue sections with the use of primary antibodies as previously described. Slides were stained for PAX8 (#ZM0468, 1:100), Ber-EP4 (#ZM0099, 1:100), CA125 (#ZM0019, 1:100), Ki67 (#ZM0166, 1:200), p16 (#ZM0205, 1:100), p53 (#ZM0408, 1:50) and WT1 (#ZM0269, 1:100) all from OriGen Technologies, Inc. Anti-CD8 (66868-1-Ig, 1:2000) and anti-CD4 (67786-1-Ig, 1:1000) were purchased from Proteintech. Images were captured using a digital scanning microscopic imaging system (OCUS).

### Survival analysis using bulk RNA-seq data

The TCGA bulk RNA-seq data along with survival data of human high-grade serous ovarian cancer patients were downloaded from UCSC Xena (https://xenabrowser.net/datapages/). For evaluating the effects of signature genes of specific clusters on survival, Z-scores were first calculated for the mean expression of the signature genes. The point with the most significant split, as determined using a log-rank test, was defined as the optimal cutoff. The Kaplan-Meier survival curves were plotted using the R package survminer and the statistical analysis was determined using the R package survival.

### Statistical analysis

For the single-cell data analysis, the association between two variables including samples, clones, and genes was assessed by Pearson correlation analysis. Wilcox Rank-sum test was used to compare the differentially expressed marker genes. Fisher’s Exact test was used for pathway significant analysis. Benjamini & Hochberg correction was performed for multiple comparisons.

## Supplementary information

Supplemental information

## Data Availability

Single-cell RNA sequencing data are available: GEO Series accession number GSE191301.
