# Supplementary material for: The impact of neoadjuvant chemotherapy on the tumor microenvironment in advanced high-grade serous carcinoma
Source: Oncogenesis. 2022 Jul 30;11(1):43. doi: 10.1038/s41389-022-00419-1 (PMC9338965; doi:10.1038/s41389-022-00419-1)
Supplement: Supplementary file 1 — Supplemental information [file 41389_2022_419_MOESM1_ESM.pdf]

The impact of neoadjuvant chemotherapy on the tumor microenvironment in advanced high-grade serous carcinoma

Yuanming Shen [1, †], Yan Ren [2 †], Kelie Chen [1 †], Yixuan Cen [2], Bo Zhang [3], Weiguo Lu[1, 2], Junfen Xu [4, 2, 1, *]

[1] Department of Gynecologic Oncology, Women's Hospital, Zhejiang University School of Medicine, Hangzhou, Zhejiang, China

[2] Women's Reproductive Health Laboratory of Zhejiang Province, Women's Hospital, Zhejiang University School of Medicine, Hangzhou, Zhejiang, China

[3] Novel Bioinformatics Co., Ltd, Shanghai, China

[4] Zhejiang Provincial Key Laboratory of Precision Diagnosis and Therapy for Major Gynecological Diseases, Women's Hospital, Zhejiang University School of Medicine, Hangzhou, Zhejiang, China.

[†] These authors contributed equally to this work.

[*] Correspondence should be addressed to Junfen Xu, xjfzu@zju.edu.cn.

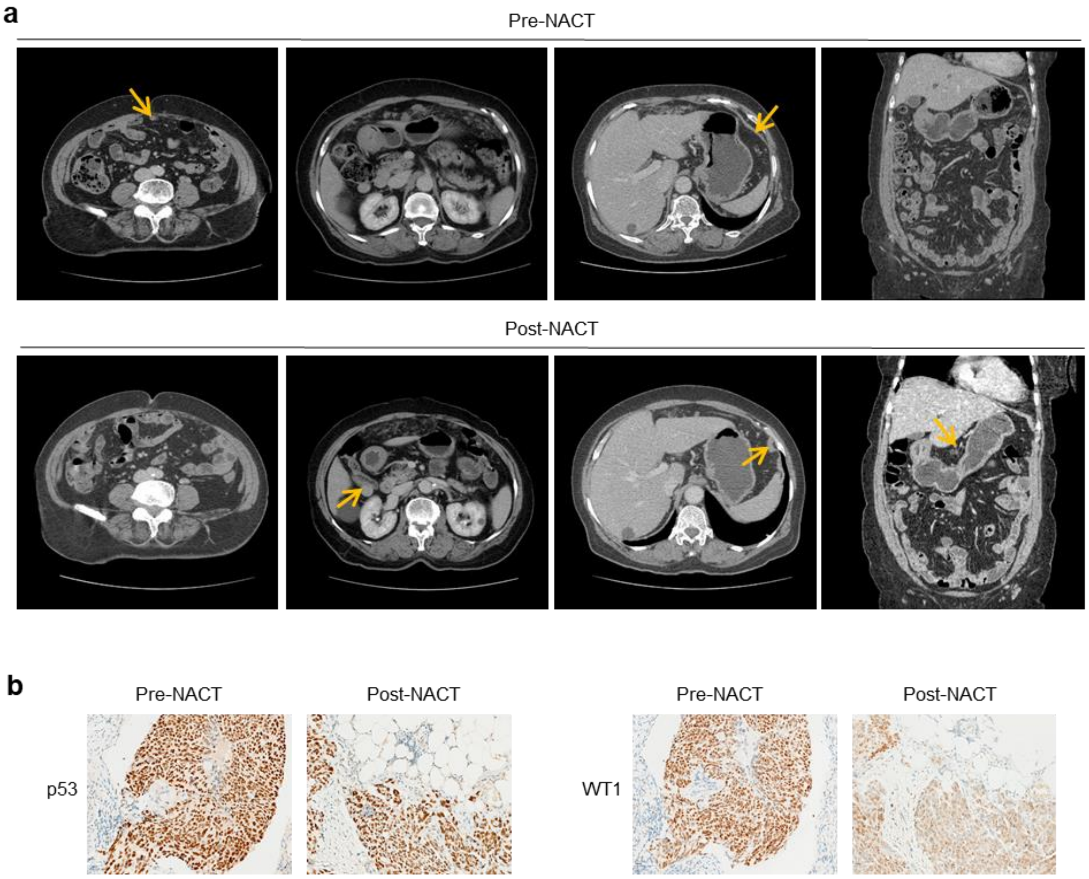

Figure S1. Features of an advanced-stage high-grade serous fallopian tube carcinoma. (a) CT imaging showing the sample acquisition for scRNA-seq in the patient with high-grade serous fallopian tube carcinoma in response to NACT . (b) IHC staining of P53 and WT1 protein in pre-NACT and post-NACT tissue samples of the high-grade serous fallopian tube carcinoma.

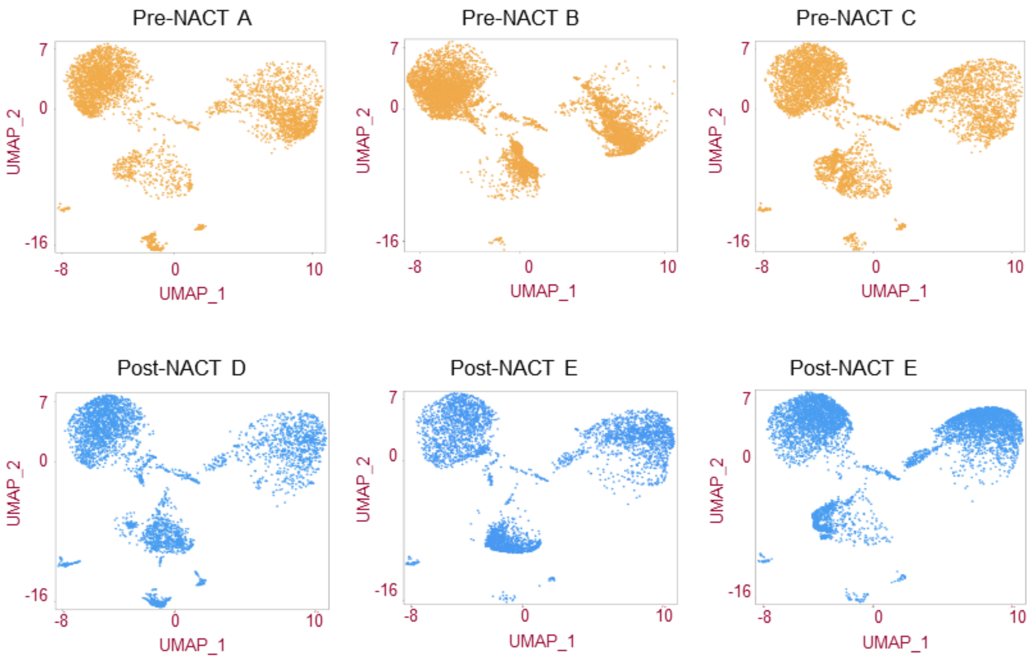

Figure S2. UMAP plots showing cell distribution in each pre-NACT and post-NACT sample.

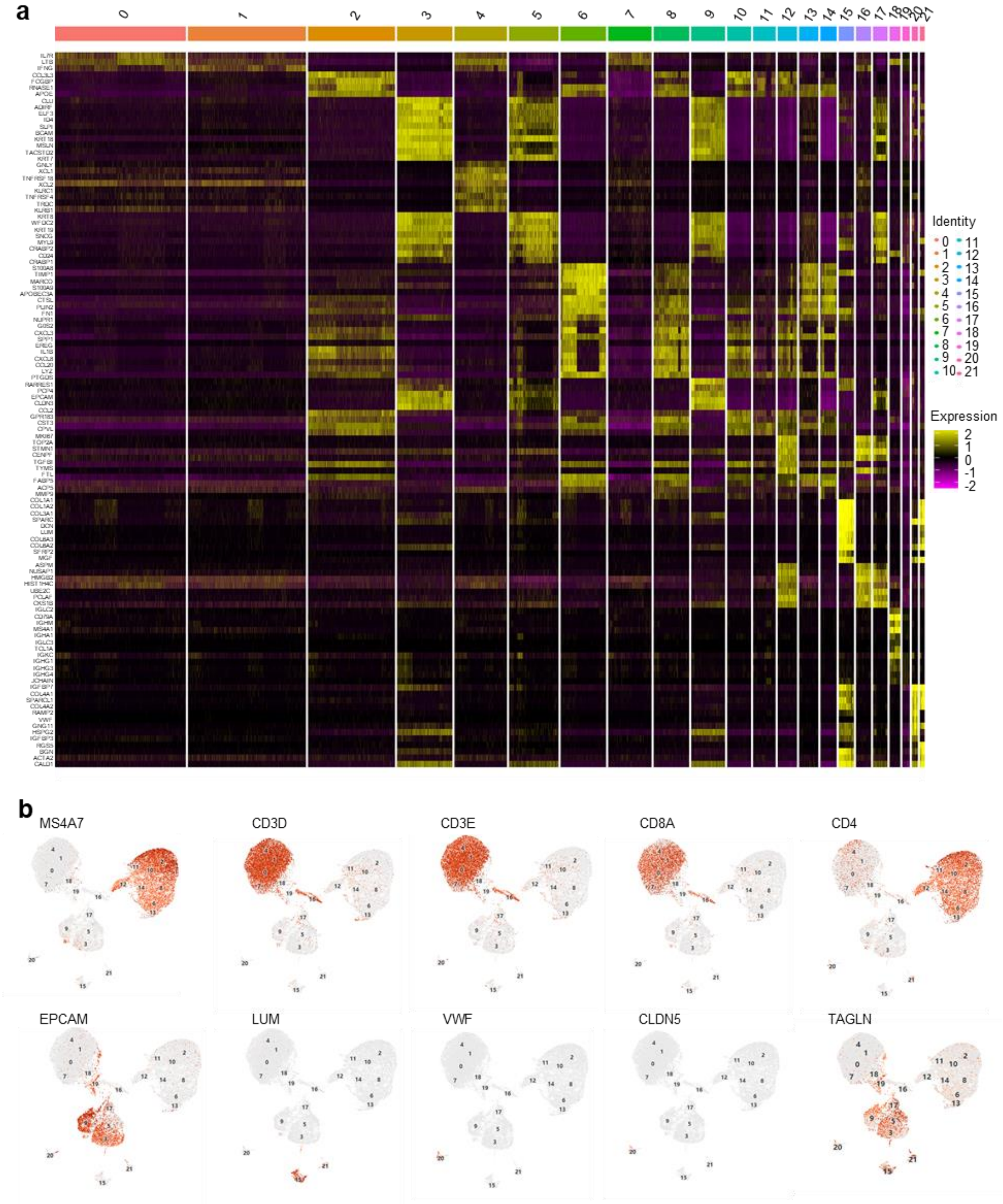

Figure S3. Features of the pre-NACT and post-NACT high-grade serous fallopian tube carcinoma samples. (a) Heatmap of the cell clusters with unique signature genes. (b) UMAP plots color-coded for expression (grey to orange) of marker genes for the cell types.

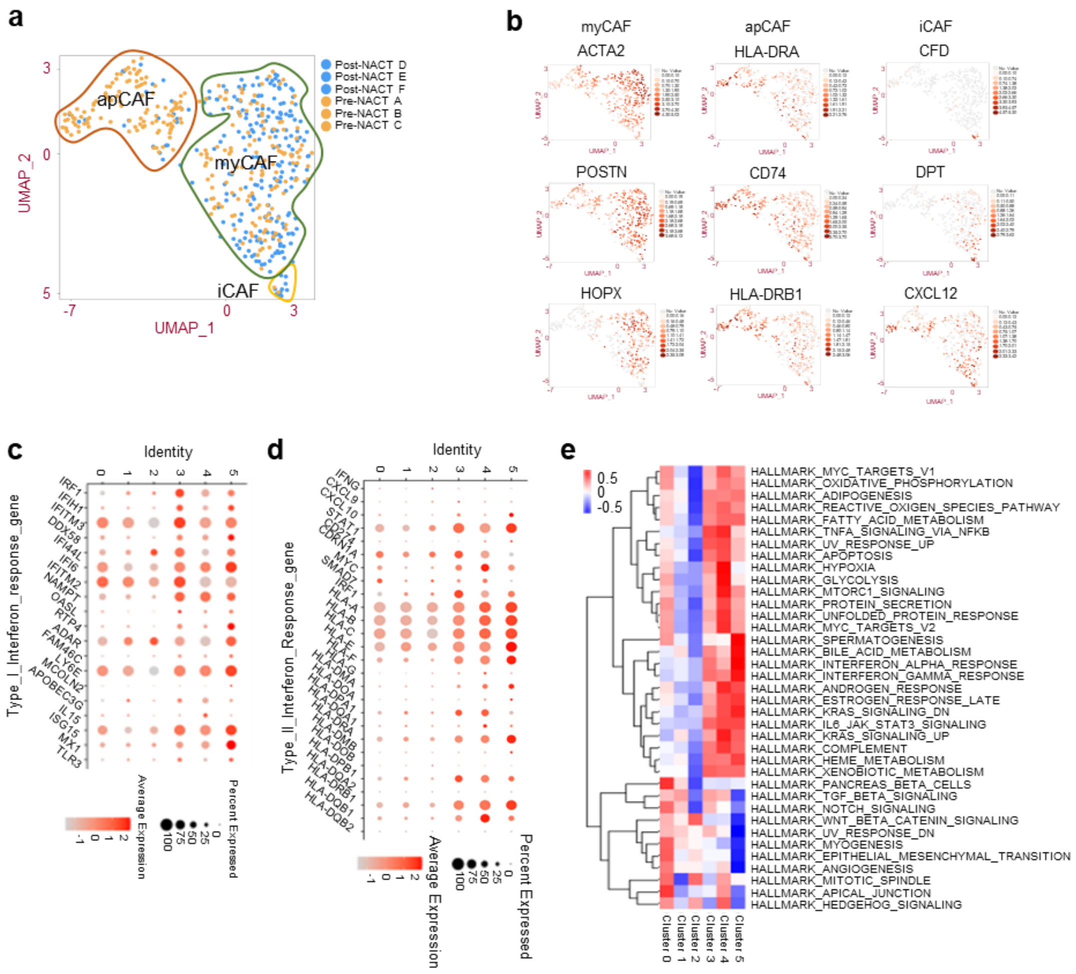

Figure S4. The subtypes of CAFs in pre-NACT and post-NACT tumors. (a) UMAP plots showing the color-coded cell subtypes of CAFs. (b) UMAP plots color-coded for the expression (gray to orange) of marker genes for the distinct cell subtyptes of CAFs. (c) Dot plots showing the expression levels of specific type I interferon response genes in each CAF subcluster. (d) Dot plots showing the expression levels of specific type II interferon response genes in each CAF subcluster. (e) Heatmap showing differentially activated hallmark pathways of each CAF subcluster.

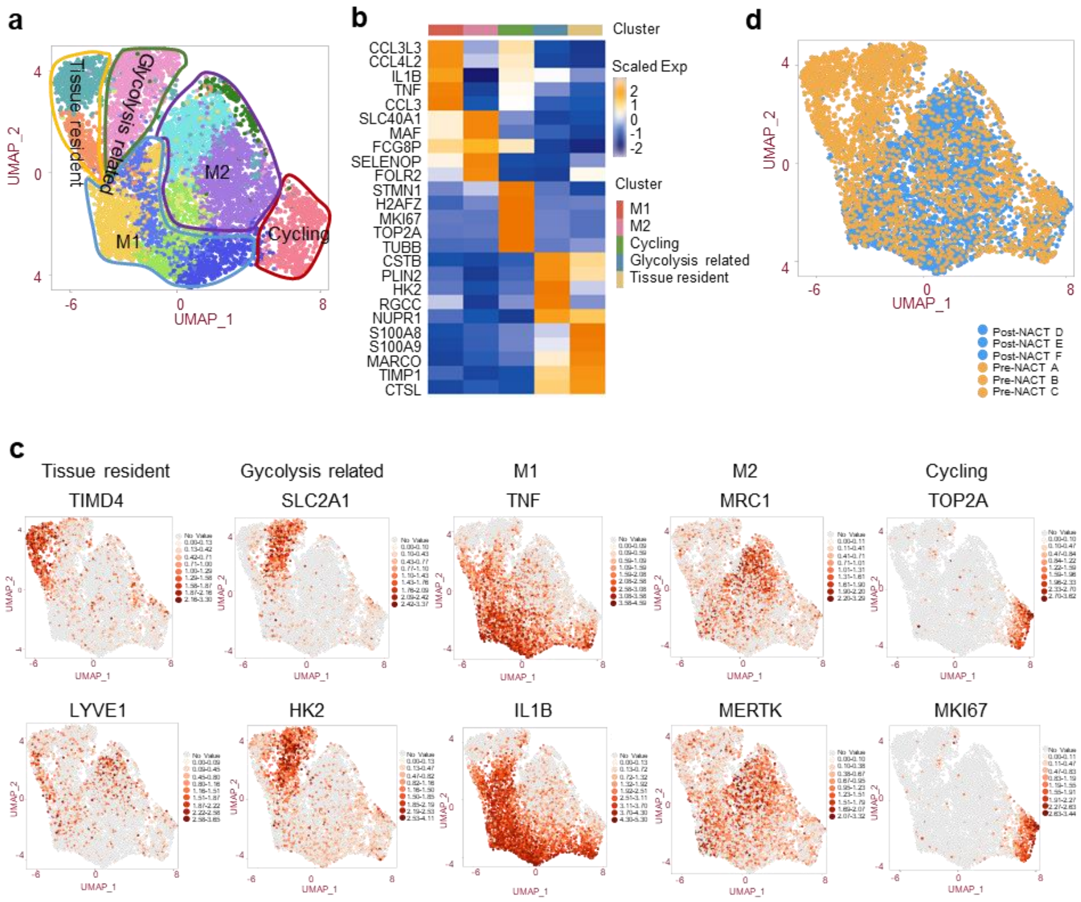

Figure S5. Macrophage cell components in pre-NACT and post-NACT tumors. (a) UMAP plots showing the subtypes of macrophages from pre-NACT and post-NACT tumors. Subtype annotations are indicated in the figure. (b) Heatmap depicting the marker gene enrichment for each cell subtype of macrophages. (c) UMAP plots showing the expression of specific marker genes in distinct macrophage subtypes. (d) UMAP plots showing pre-NACT and post-NACT macrophages, clustered and color-coded according to the group.

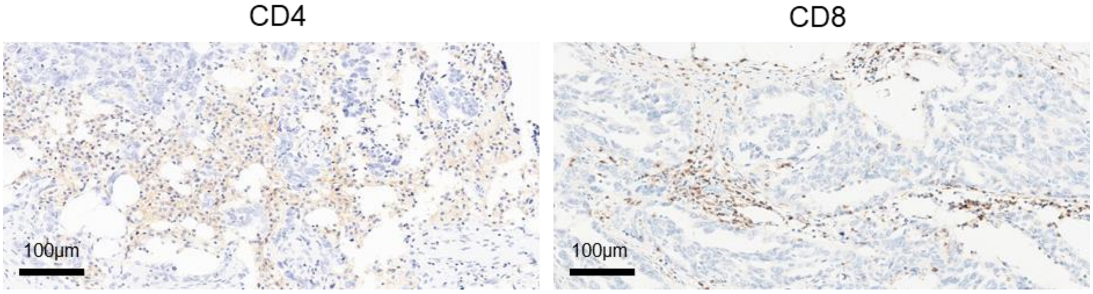

Figure S6. The IHC staining of CD4 and CD8 in post-NACT tumors. The representative CD4 and CD8 IHC staining showing the expression and location of $CD4^+$ and $CD8^+$ T cells in post-NACT tumors. Scale bar: 100 μm.

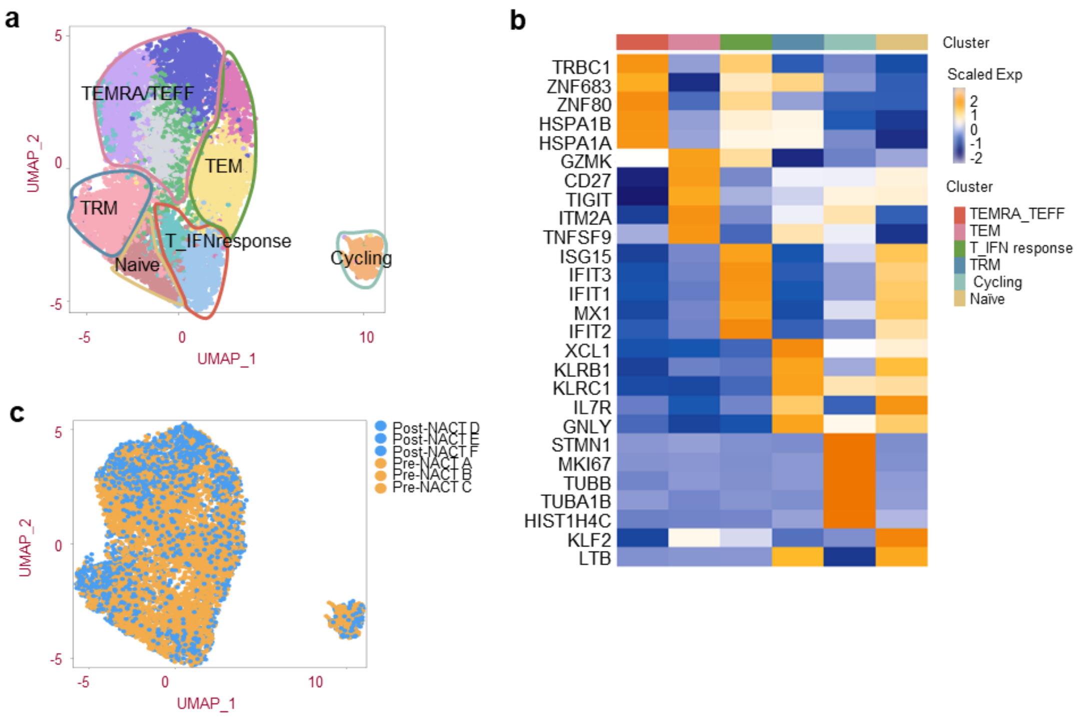

Figure S7. The subtypes of CD8$^+$ T cells in pre-NACT and post-NACT tumors. (a) UMAP plots showing the subtypes of CD8$^+$ T cells from pre-NACT and post-NACT tumors. Subtype annotations are indicated in the figure. (b) Heatmap depicting the marker gene enrichment for each cell subtype of CD8$^+$ T cells. (c) UMAP plots showing the color-coded cell groups of CD8$^+$ T cells in response to NACT.

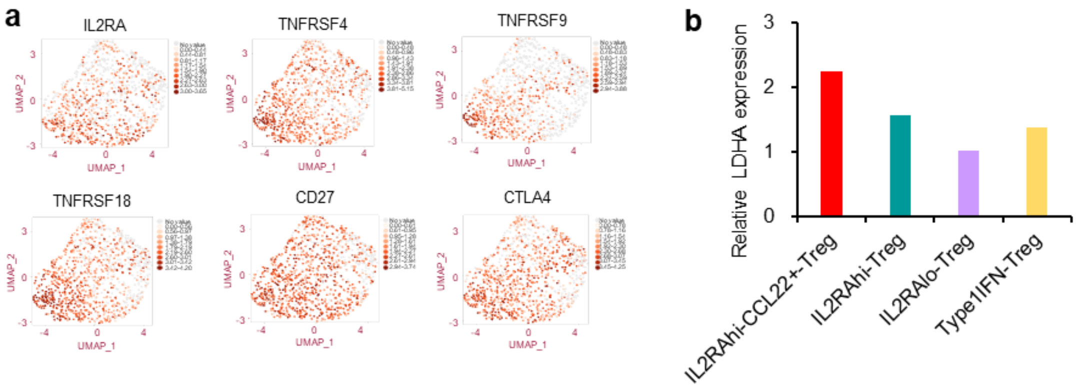

Figure S8. Features of Treg subpopulations in pre-NACT and post-NACT tumors. (a) UMAP plots showing the expression of specific immune checkpoint genes in Treg cells. (b) the relative expression level of LDHA in each Treg subpopulation.

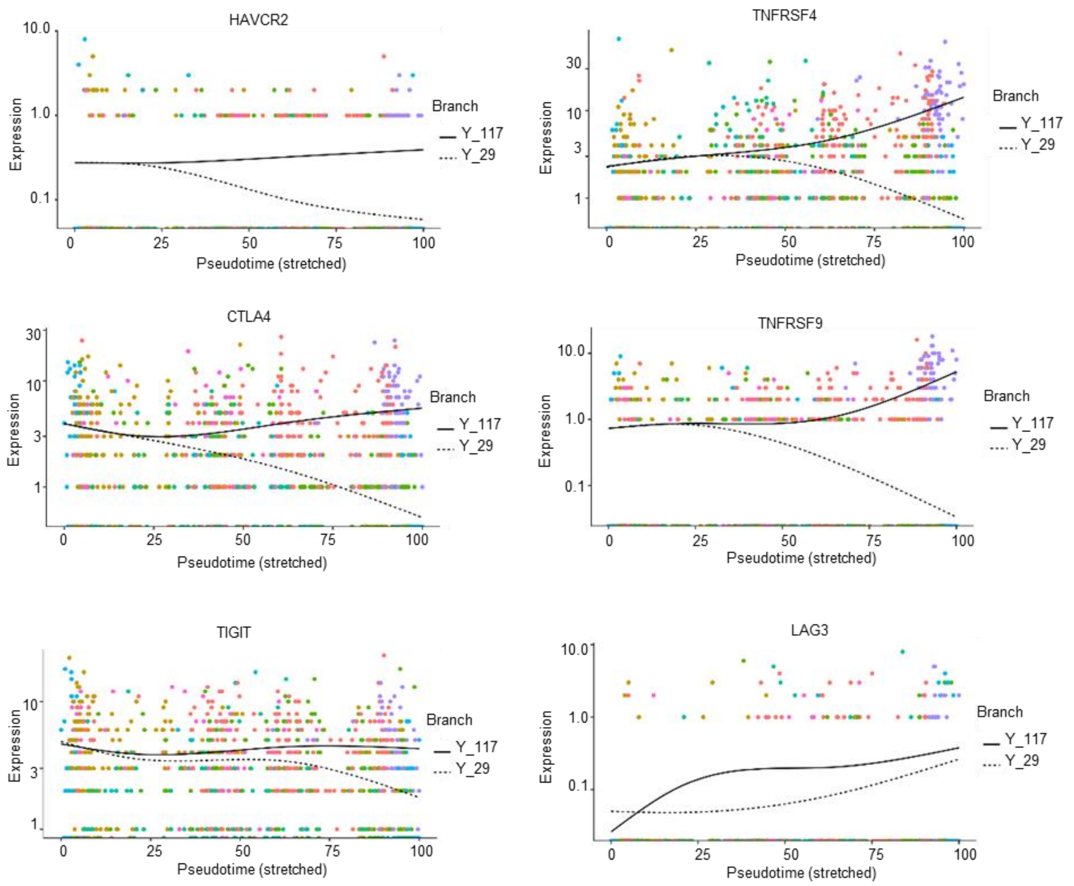

Figure S9. Analysis of the expression levels of beam genes for IL2RAhi-CCL22+-Treg cells. Two-dimensional plots showing the expression scores for specific beam genes during IL2RAhi-CCL22+-Treg cell transitions, along with the pseudotime.

Table S1. Maker gene signature in epithelial clone 63 cells.

marker genes	p_val_adj	avg_logFC	marker genes	p_val_adj	avg_logFC
PTGDS	6.9007E-226	1.563918221	LAPTM4B	0	0.989211921
PCP4	0	1.529557182	CCDC146	0	0.986435344
BCAM	0	1.449697808	THY1	0	0.984780629
CLDN3	0	1.438242301	CD24	3.8095E-252	0.976435917
MSLN	0	1.398854497	KLK5	0	0.97459407
CLDN4	0	1.348271768	C19orf33	0	0.971085531
LCN2	0	1.325698429	RBP1	0	0.940374518
CTGF	0	1.32288153	TFPI2	0	0.937603081
EPCAM	0	1.31593379	FHL2	0	0.934993968
TM4SF1	0	1.229208655	DSP	0	0.933653279
CYR61	0	1.193450401	PLAT	0	0.930374699
SNCG	0	1.192083733	LY6G6C	0	0.928376619
S100A1	0	1.16989215	SCARA3	0	0.928255187
CRYAB	0	1.150138985	TNNT2	0	0.912744535
MUC16	0	1.146431937	IGFBP2	6.3422E-295	0.909203086
IGF2	3.3642E-272	1.130272587	KLK7	0	0.904830122
C1orf186	0	1.094856701	GPRC5A	0	0.893447031
PAWR	0	1.070744118	TJP1	0	0.891006403
ID1	3.6711E-303	1.067309106	C9orf3	0	0.870284058
KCNK15	0	1.061008327	MAL2	0	0.864323545
RARRES1	5.8379E-240	1.053671757	RAB25	0	0.864174903
MFGE8	0	1.035136898	DMKN	0	0.849144918
CITED4	0	1.026448824	NMU	0	0.84510106
EMP2	0	1.021825502	C20orf204	0	0.834735652
AKAP12	0	1.01439669	PHGDH	0	0.827717522
NR2F6	0	1.00524175	PDZK1IP1	0	0.823102957
S100A14	0	0.996069623	FXYD3	0	0.818172787
TIMP3	0	0.990246888	CTXN1	0	0.813584684
